# Crystal structure of *N*,*N*′-bis­[3-(methyl­sulfan­yl)prop­yl]-1,8:4,5-naphthalene­tetra­carb­oxy­lic di­imide

**DOI:** 10.1107/S2056989019007771

**Published:** 2019-05-31

**Authors:** Juhyeon Park, Seung Heon Lee, Myong Yong Choi, Cheol Joo Moon, Tae Ho Kim

**Affiliations:** a Department of Chemistry (BK21 plus) and Research Institute of Natural Sciences, Gyeongsang National University, Jinju 52828, Republic of Korea

**Keywords:** crystal structure, naphthalene­tetra­carb­oxy­lic di­imide, crystal packing, hydrogen bonding, DFT calculations, Hirshfeld surface analysis.

## Abstract

The title compound, C_22_H_22_N_2_O_4_S_2_, was synthesized by the reaction of 1,4,5,8-naphthalene­tetra­carb­oxy­lic dianhydride with 3-(methyl­sulfan­yl)propyl­amine. The whole mol­ecule is generated by an inversion operation of the asymmetric unit. This mol­ecule has an *anti* form with the terminal methyl­thio­propyl groups above and below the aromatic di­imide plane, where four intra­molecular C—H⋯O and C—H⋯S hydrogen bonds are present and the O⋯H⋯S angle is 100.8°. DFT calculations revealed slight differences between the solid state and gas phase structures. In the crystal, C—H⋯O and C—H⋯S hydrogen bonds link the mol­ecules into chains along the [2 direction. adjacent chains are inter­connected by π–π inter­actions, forming a two-dimensional network parallel to the (001) plane. Each two-dimensional layer is further packed in an *ABAB* sequence along the *c*-axis direction. Hirshfeld surface analysis shows that van der Waals inter­actions make important contributions to the inter­molecular contacts. The most important contacts found in the Hirshfeld surface analysis are H⋯H (44.2%), H⋯O/O⋯H (18.2%), H⋯C/C⋯H (14.4%), and H⋯S/S⋯H (10.2%).

## Abstract 2

The title compound was synthesized from naphthalene di­imide and methyl­thio­propi­amine. The asymmetric unit consists of half of the total mol­ecule as the mol­ecule lies on an inversion center. Intra­molecular C—H⋯O and C—H⋯S hydrogen bonds cause the mol­ecule to have an *anti* conformation. In the crystal, C—H⋯O and C—H⋯S hydrogen bonds and π–π inter­actions lead to the formation of a two-dimensional network structure parallel to (110). Hirshfeld surface analysis confirmed the inter­molecular inter­actions.

## Chemical context   

Naphthalene di­imide, which has an expanded π-electron-deficient plane has attracted considerable inter­est as an excellent organic linker material for the production of photochromic coordination polymers as a result of their photoinduced electron transfer from neutral organic moieties to stable anionic radicals (Liu *et al.*, 2018 ▸). Aromatic imides are highly fluorescent residues that are used in the signal generation of sensors or on–off mol­ecular switches. They have also been used in the design of receptors (Claudio-Catalán *et al.*, 2016 ▸) and sensors to recognize charged species and other guests (Landey-Álvarez *et al.*, 2016 ▸). In addition, naphthalene di­imides are ideal for studying anionic⋯π inter­actions because the quadrupole moments are highly positive (Fang *et al.*, 2015 ▸). We have extended our work on naphthalene di­imides to produce the title compound by the reaction of naphthalene­carb­oxy­lic dianhydride with methyl­thio­pyrimidine and report its crystal structure here.
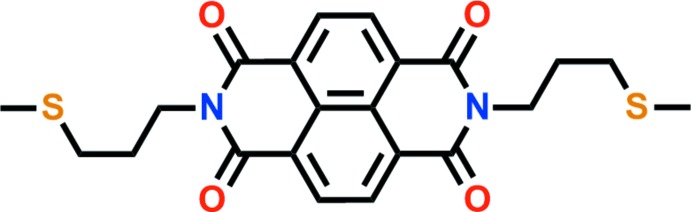


## Structural commentary   

**Figure 1:**
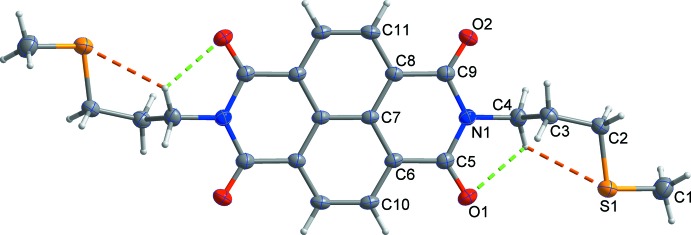
The asymmetric unit of the title compound, with displacement ellipsoids drawn at the 50% probability level. H atoms are shown as small spheres of arbitrary radius and yellow and green dashed lines represent intra­molecular C—H⋯S and C—H⋯O hydrogen bonds, respectively. Unlabelled atoms are generated by the symmetry operation (−*x* + 2, −*y* + 1, −*z*).

The title compound comprises a central naphthalene di­imide with terminal thio­propyl chains (Fig. 1 ▸). The mol­ecule lies on a crystallographic inversion center located at the centroid of the naphthalene ring system and the asymmetric unit is composed of one half of the mol­ecule. As expected, the naphthalene di­imide plane (N1/C5/O1/C6/C10/C7/C8/C11/C9/O2) is roughly planar with an r.m.s. deviation of 0.024 Å. The total distance between the terminal carbon atoms is 18.621 Å. Furthermore, this mol­ecule has an *anti* form as a result of the intra­molecular C4—H4*A*⋯O1 and C4—H4*A*⋯S1 hydrogen bonds (Table 1 ▸). The terminal methyl­thio­propyl­amine group is fixed at an O1⋯H4*A*⋯S1 angle of 100.8° by the aforementioned intra­molecular hydrogen bonds. The C3/C2/S1/C1 section of the methyl­thio­propyl substituent is almost parallel to the naphthalene di­imide unit with the C3/C2/S1/C1 mean plane inclined to the naphthalene di­imide plane at a dihedral angle of 13.1 (2)°.

## Theoretical calculations   

**Figure 2:**
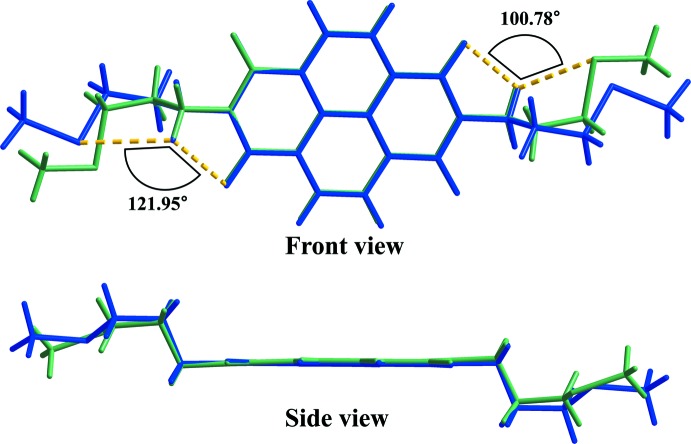
Atom-by-atom superimposition of the calculated structure (blue) using B3LYP/6–311^++^G** and the X-ray structure (green) for the title compound.

DFT calculations were performed using the *GAUSSIAN09* software package (Frisch *et al.*, 2009 ▸) and the calculated distances and angles were compared with experimental values from the X-ray diffraction studies. The overall structural calculation was performed using the B3LYP level theory with a 6–311^++^G** basis set. The parameters optimized for bond lengths and bond angles are in close agreement with experimental crystallographic data (Table 2 ▸). The terminal methyl­thio­propyl group is fixed by inter­nal hydrogen bonding in the crystal, whereas its inter­nal hydrogen bonds are broken in the gas-phase structural calculation. This can be confirmed by the fact that the O1⋯H4*A*⋯S1 angle of the methyl­thio­propyl group has changed from 100.8 to 122.0° (Fig. 2 ▸). However, even in the gas phase the mol­ecule has an *anti* form similar to that found in the solid state.

## Supra­molecular features   

**Figure 3:**
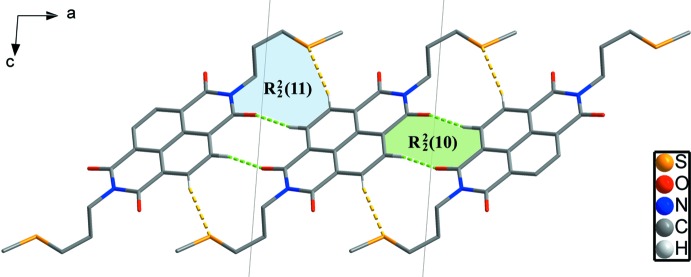
Inter­molecular C—H⋯S and C—H⋯O hydrogen bonds (yellow and green dashed lines) forming chains along the [2
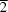
0] direction with 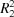
(11) and 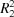
(10) motifs.

**Figure 4:**
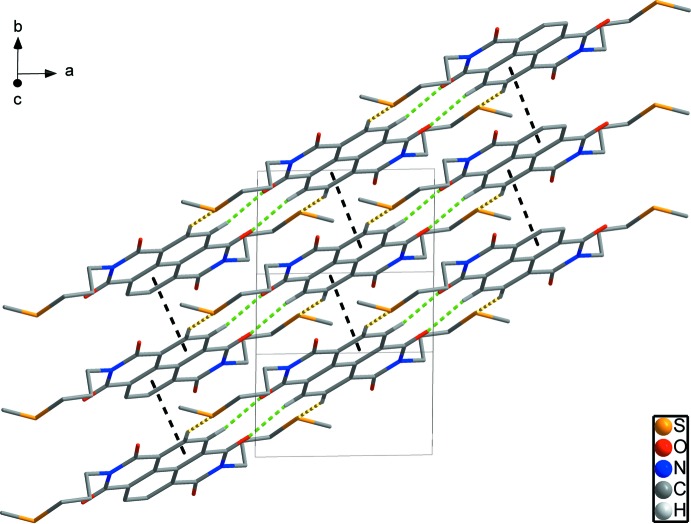
A packing diagram for the title compound, showing the two-dimensional network formed by C—H⋯S and C—H⋯O hydrogen bonds (yellow and green dashed lines) and π–π inter­actions (black dashed lines). H atoms not involved in inter­molecular inter­actions have been omitted for clarity.

**Figure 5:**
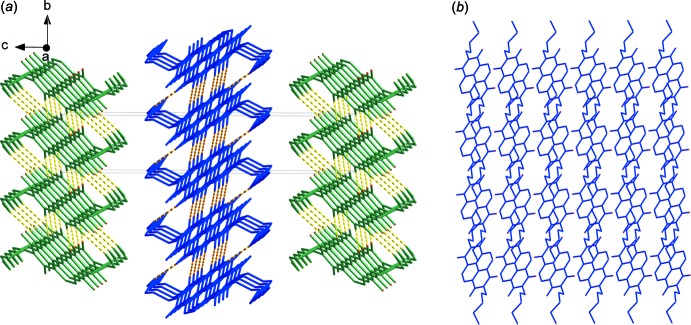
Packing diagrams of the title compound showing (*a*) the *ABAB* stacking pattern and (*b*) the two-dimensional structure.

In the crystal, C—H⋯O and C—H⋯S hydrogen bonds (Table 1 ▸) link the mol­ecules, forming 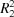
(11) and 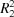
(10) rings (Fig. 3 ▸) and resulting in chains along the [2
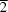
0] direction. Adjacent chains are linked by inter­molecular π–π inter­actions between naphthalene di­imide rings [*Cg*1⋯*Cg*2 = 3.5756 (12) Å; *Cg*1 and *Cg*2 are the centroids of the C6/C7/C7^iii^/C8^iii^/C10/C11^iii^ and C6^iv^/C7^iv^/C7^v^/C8^v^/C10^iv^/C11^v^ rings, respectively; symmetry codes: (iii) −*x* + 2, −*y* + 1, −*z*; (iv) −*x* + 2, −*y*, −*z*; (v) *x*, *y* − 1, *z*]. These π–π inter­actions lead to a two-dimensional network structure parallel to the (001) plane (Fig. 4 ▸). The network structures are stacked in an alternating *ABAB* sequence along the *c*-axis direction (Fig. 5 ▸).

## Hirshfeld surface analysis   

**Figure 6:**
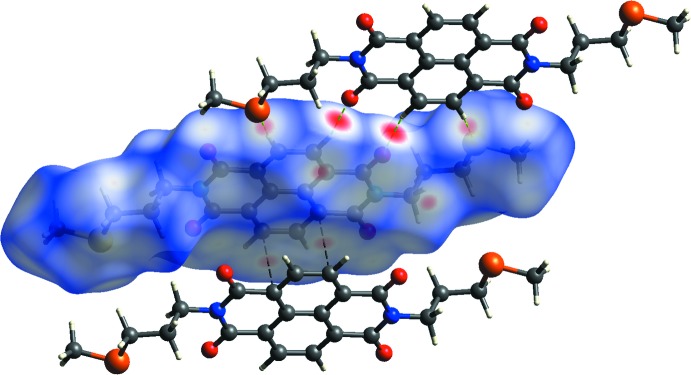
A view of the Hirshfeld surface of the title compound mapped over *d*_norm_, showing the H⋯S and H⋯O contacts of the inter­molecular inter­actions using a fixed colour scale of −0.2580 (red) to 1.0789 (blue) a.u.

Hirshfeld surface analysis was performed using *CrystalExplorer* (Turner *et al.*, 2017 ▸) to qu­antify the various inter­molecular inter­actions in the mol­ecular packing of the title compound. The bright red dots in Fig. 6 ▸ showing the Hirshfeld surface mapped to the normalized contact distance (*d*_norm_) indicate the 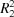
(11) and 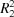
(10) loops, and the contact points of the inter­molecular C—H⋯O and C—H⋯S hydrogen bonds. The lighter red dot on the surface represents the π–π inter­action with adjacent mol­ecules. The white and blue colours that make up the majority of the surface indicate contact distances that are equal to or greater than the van der Waals radii.

**Figure 7:**
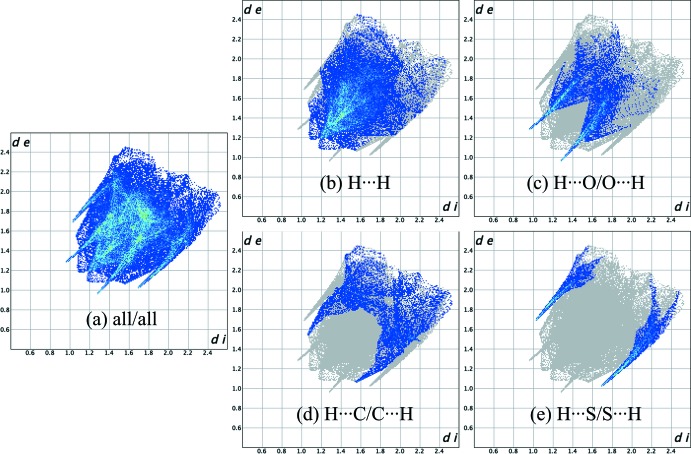
(*a*) The full two-dimensional fingerprint plot for the title compound and those delineated into (*b*) H⋯H, (*c*) H⋯O/O⋯H, (*d*) H⋯C/C⋯H and (*e*) H⋯S/S⋯H contacts. The *d*_i_ and *d*_e_ values are the closest inter­nal and external distances (in Å) from given points on the Hirshfeld surface contacts.

The C—H⋯O and C—H⋯S hydrogen bonds and π–π stacking inter­actions are identified in the two-dimensional fingerprint plots (Fig. 7 ▸ *a*–*e*), which show the H⋯H, H⋯C/C⋯H, H⋯O/ O⋯H, H⋯N/N⋯H, and H⋯S/S⋯H contacts. The relative contributions of the atomic contacts to the Hirshfeld surface are summarized in Table 3 ▸. These show that the dominant inter­action, accounting for 44.2% of the surface, is the H⋯H van der Waals inter­action. Substantial contributions are also made by H⋯O/O⋯H (18.3%), H⋯C/C⋯H (14.4%), and H⋯S/S⋯H (10.2%) contacts, which are indicated by two sharp peaks in each fingerprint plot. Lesser contributions from C⋯O/O⋯C, C⋯C, H⋯N/N⋯H, O⋯O, N⋯O/O⋯N, and C⋯S/S⋯C contacts are included in Table 3 ▸ for completeness.

## Synthesis and crystallization   

A mixture of 1,4,5,8-naphthalene­tetra­carb­oxy­lic dianhydride (6.70 g, 25.0 mmol) and 3-(methyl­sulfan­yl)propyl­amine (5.6 mL, 50.0 mmol) in toluene (5 mL) and quinoline (15 mL) was heated at 453 K with stirring for 1h. Upon cooling to room temperature, a golden yellow crude solid was filtered off and washed with diethyl ether. A golden yellow powder was obtained. Crystals suitable for X-ray diffraction analysis were obtained by slow evaporation of a di­chloro­methane solution of the title compound.

^1^H NMR (300 MHz, CDCl_3_): δ 8.77 (*s*, 2H, Ar), 4.33 (*t*, 2H, CH_2_N), 2.64 (*t*, 2H, CH_2_), 2.14 (*s*, 3H, CH_3_), 2.07 (*t*, 2H, CH_2_S). ^13^C NMR (75.4 MHz, CDCl_3_): δ 162.81, 130.99, 126.69, 126.57, 40.01, 31.61, 27.16 and 15.31. IR (ν, cm^−1^): 3344 (*m*); 3071 (*m*); 2916 (*s*); 2848 (*s*); 1999 (*s*); 1693 (*s*).

## Database survey   

A search of the Cambridge Structural Database (CSD, Version 5.40, updated February 2019; Groom *et al.*, 2016 ▸) for naphthalene di­imide derivatives gave 31 hits for structures that include a terminal propyl group. The title compound was not found. Related compounds include a series of cyclo­alkyl-substituted naphthalene tetra­carb­oxy­lic di­imides (Kakinuma *et al.*, 2013 ▸). Other terminal *n*-alkyl groups are known with 2,7-di­butyl­benzo[*lmn*][3,8]phenanthroline-1,3,6,8-tetra­one (Alvey *et al.*, 2010 ▸), bis-*N*,*N*′-di­pentyl­naphthalene-1,4,5,8-tetra­carb­oxy­lic di­imide (Andric *et al.*, 2004 ▸), *N*,*N*′-di-*n*-hexyl-1,4;5,8-naphthalene­tetra­carb­oxy­lic di­imide (Ofir *et al.*, 2006 ▸), and *N*,*N*′-di(*n*-dodec­yl)naphthalene-4,5,8,9-tetra­carb­oxy­lic acid di­imide (Kozycz *et al.*, 2015 ▸).

## Refinement   

Crystal data, data collection and structure refinement details are summarized in Table 4 ▸. All H atoms were positioned geometrically and refined using a riding model with *d*(C—H) = 0.95 Å, *U*_iso_ = 1.2*U*_eq_(C) for aromatic, *d*(C—H) = 0.99 Å, *U*_iso_ = 1.2*U*_eq_(C) for methyl­ene, and *d*(C—H) = 0.98 Å, *U*_iso_ = 1.5*U*_eq_(C) for the methyl H atoms.

## Supplementary Material

Crystal structure: contains datablock(s) I, New_Global_Publ_Block. DOI: 10.1107/S2056989019007771/sj5571sup1.cif

Structure factors: contains datablock(s) I. DOI: 10.1107/S2056989019007771/sj5571Isup2.hkl

CCDC reference: 1919395

Additional supporting information:  crystallographic information; 3D view; checkCIF report

## Figures and Tables

**Table 1:** Hydrogen-bond geometry (Å, °)

*D*—H⋯*A*	*D*—H	H⋯*A*	*D*⋯*A*	*D*—H⋯*A*
C4—H4*A*⋯S1	0.99	2.84	3.300 (2)	109
C4—H4*A*⋯O1	0.99	2.32	2.688 (3)	101
C10—H10⋯O1^i^	0.95	2.39	3.316 (3)	164
C11—H11⋯S1^ii^	0.95	2.86	3.779 (2)	162

Symmetry codes: (i) 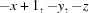; (ii) 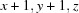.

**Table 2:** Experimental and calculated bond lengths (Å)

Bond	X-ray	B3LYP (6–311++G**)
O1—C5	1.210 (3)	1.2158
O2—C9	1.221 (3)	1.2173
N1—C4	1.471 (3)	1.4779
N1—C5	1.404 (3)	1.4047
N1—C9	1.394 (3)	1.4029
S1—C1	1.785 (3)	1.8244
S1—C2	1.803 (2)	1.8399
C2—C3	1.523 (3)	1.5301
C3—C4	1.521 (3)	1.5341
C5—C6	1.479 (3)	1.4880
C6—C7	1.408 (3)	1.4135
C7—C8	1.415 (3)	1.4136
C8—C9	1.478 (3)	1.4877
C6—C10	1.381 (3)	1.3835
C8—C11	1.373 (3)	1.3835

**Table 3:** Percentage contributions of inter­atomic contacts to the Hirshfeld surface of the title compound.

Contact	Percentage contribution
H⋯H	44.2
H⋯O/O⋯H	18.3
H⋯C/C⋯H	14.4
H⋯S/S⋯H	10.2
C⋯O/O⋯C	5.6
C⋯C	4.5
H⋯N/N⋯H	1.4
O⋯O	0.5
N⋯O/O⋯N	0.4
C⋯S/S⋯C	0.4

**Table 4:** Experimental details

Crystal data	
Chemical formula	C_22_H_22_N_2_O_4_S_2_
*M*_r_	442.53
Crystal system, space group	Monoclinic, *P*2_1_/*c*
Temperature (K)	173
*a*, *b*, *c* (Å)	8.0500 (2), 4.9407 (1), 24.9626 (7)
β (°)	94.333 (2)
*V* (Å^3^)	989.99 (4)
*Z*	2
Radiation type	Mo *K*α
μ (mm^−1^)	0.30
Crystal size (mm)	0.23 × 0.05 × 0.04

Data collection	
Diffractometer	Bruker APEXII CCD
Absorption correction	Multi-scan (*SADABS*; Bruker, 2014 ▸)
*T*_min_, *T*_max_	0.676, 0.746
No. of measured, independent and observed [*I* > 2σ(*I*)] reflections	5641, 1732, 1360
*R*_int_	0.046
(sin θ/λ)_max_ (Å^−1^)	0.595

Refinement	
*R*[*F* ^2^ > 2σ(*F* ^2^)], *wR*(*F* ^2^), *S*	0.042, 0.101, 1.05
No. of reflections	1732
No. of parameters	137
H-atom treatment	H-atom parameters constrained
Δρ_max_, Δρ_min_ (e Å^−3^)	0.25, −0.26

Computer programs: *APEX2* and *SAINT* (Bruker, 2014 ▸), *SHELXS97* and *SHELXTL* (Sheldrick, 2008 ▸), *SHELXL2014* (Sheldrick, 2015 ▸), *DIAMOND* (Brandenburg, 2010 ▸) and *publCIF* (Westrip, 2010 ▸).

## supplementary crystallographic information

### Crystal data

**Table:**

C_22_H_22_N_2_O_4_S_2_	*F*(000) = 464
*M**_r_* = 442.53	*D*_x_ = 1.485 Mg m^−^^3^
Monoclinic, *P*2_1_/*c*	Mo *K*α radiation, λ = 0.71073 Å
*a* = 8.0500 (2) Å	Cell parameters from 1676 reflections
*b* = 4.9407 (1) Å	θ = 3.1–26.1°
*c* = 24.9626 (7) Å	µ = 0.30 mm^−^^1^
β = 94.333 (2)°	*T* = 173 K
*V* = 989.99 (4) Å^3^	Rod, yellow
*Z* = 2	0.23 × 0.05 × 0.04 mm

### Data collection

**Table:**

Bruker APEXII CCD diffractometer	1360 reflections with *I* > 2σ(*I*)
φ and ω scans	*R*_int_ = 0.046
Absorption correction: multi-scan (SADABS; Bruker, 2014)	θ_max_ = 25.0°, θ_min_ = 1.6°
*T*_min_ = 0.676, *T*_max_ = 0.746	*h* = −9→9
5641 measured reflections	*k* = −5→5
1732 independent reflections	*l* = −29→25

### Refinement

**Table:**

Refinement on *F*^2^	0 restraints
Least-squares matrix: full	Hydrogen site location: inferred from neighbouring sites
*R*[*F*^2^ > 2σ(*F*^2^)] = 0.042	H-atom parameters constrained
*wR*(*F*^2^) = 0.101	*w* = 1/[σ^2^(*F*_o_^2^) + (0.0403*P*)^2^ + 0.3426*P*] where *P* = (*F*_o_^2^ + 2*F*_c_^2^)/3
*S* = 1.05	(Δ/σ)_max_ < 0.001
1732 reflections	Δρ_max_ = 0.25 e Å^−^^3^
137 parameters	Δρ_min_ = −0.26 e Å^−^^3^

### Special details

**Table:**

Geometry. All esds (except the esd in the dihedral angle between two l.s. planes) are estimated using the full covariance matrix. The cell esds are taken into account individually in the estimation of esds in distances, angles and torsion angles; correlations between esds in cell parameters are only used when they are defined by crystal symmetry. An approximate (isotropic) treatment of cell esds is used for estimating esds involving l.s. planes.

### Fractional atomic coordinates and isotropic or equivalent isotropic displacement parameters (Å^2^)

**Table:**

	*x*	*y*	*z*	*U*_iso_*/*U*_eq_	
S1	0.25895 (9)	0.32924 (13)	0.17605 (3)	0.0356 (2)	
O1	0.5336 (2)	0.2804 (3)	0.04996 (7)	0.0310 (4)	
O2	0.8407 (2)	0.9669 (3)	0.12693 (6)	0.0326 (5)	
N1	0.6852 (2)	0.6297 (4)	0.08708 (8)	0.0243 (5)	
C1	0.0764 (4)	0.4611 (6)	0.20299 (12)	0.0458 (8)	
H1A	0.0888	0.4506	0.2423	0.069*	
H1B	−0.0207	0.3548	0.1895	0.069*	
H1C	0.0609	0.6503	0.1919	0.069*	
C2	0.4130 (3)	0.5621 (5)	0.20506 (10)	0.0273 (6)	
H2A	0.3685	0.7488	0.2024	0.033*	
H2B	0.4374	0.5191	0.2436	0.033*	
C3	0.5728 (3)	0.5448 (5)	0.17623 (9)	0.0276 (6)	
H3A	0.6650	0.6315	0.1985	0.033*	
H3B	0.6023	0.3526	0.1710	0.033*	
C4	0.5500 (3)	0.6857 (5)	0.12200 (10)	0.0274 (6)	
H4A	0.4431	0.6269	0.1035	0.033*	
H4B	0.5434	0.8834	0.1279	0.033*	
C5	0.6592 (3)	0.4151 (4)	0.05056 (9)	0.0243 (6)	
C6	0.7912 (3)	0.3668 (4)	0.01356 (9)	0.0214 (5)	
C7	0.9374 (3)	0.5241 (4)	0.01778 (8)	0.0200 (5)	
C8	0.9613 (3)	0.7304 (4)	0.05690 (9)	0.0228 (5)	
C9	0.8287 (3)	0.7878 (4)	0.09317 (9)	0.0249 (6)	
C10	0.7698 (3)	0.1661 (4)	−0.02484 (9)	0.0234 (5)	
H10	0.6712	0.0598	−0.0273	0.028*	
C11	1.1054 (3)	0.8802 (4)	0.06017 (9)	0.0242 (6)	
H11	1.1210	1.0173	0.0868	0.029*	

### Atomic displacement parameters (Å^2^)

**Table:**

	*U*^11^	*U*^22^	*U*^33^	*U*^12^	*U*^13^	*U*^23^
S1	0.0366 (5)	0.0304 (4)	0.0400 (4)	−0.0049 (3)	0.0038 (3)	−0.0064 (3)
O1	0.0268 (11)	0.0293 (9)	0.0371 (10)	−0.0088 (8)	0.0036 (8)	−0.0010 (8)
O2	0.0372 (11)	0.0284 (9)	0.0322 (10)	−0.0041 (8)	0.0025 (8)	−0.0113 (8)
N1	0.0252 (12)	0.0200 (10)	0.0273 (11)	−0.0021 (9)	0.0009 (9)	−0.0004 (8)
C1	0.0360 (19)	0.0483 (17)	0.0541 (19)	−0.0024 (14)	0.0104 (14)	0.0018 (15)
C2	0.0320 (16)	0.0220 (12)	0.0276 (13)	−0.0002 (11)	−0.0003 (11)	−0.0003 (10)
C3	0.0292 (15)	0.0272 (13)	0.0260 (13)	0.0017 (11)	−0.0011 (11)	−0.0019 (10)
C4	0.0254 (15)	0.0255 (13)	0.0314 (14)	0.0034 (11)	0.0038 (11)	0.0005 (11)
C5	0.0275 (15)	0.0199 (12)	0.0247 (13)	−0.0006 (11)	−0.0034 (10)	0.0039 (10)
C6	0.0237 (14)	0.0150 (11)	0.0248 (13)	0.0001 (10)	−0.0031 (10)	0.0028 (9)
C7	0.0226 (14)	0.0152 (11)	0.0214 (12)	−0.0006 (10)	−0.0037 (9)	0.0028 (9)
C8	0.0253 (14)	0.0178 (11)	0.0246 (13)	−0.0015 (10)	−0.0026 (10)	0.0028 (9)
C9	0.0284 (15)	0.0211 (12)	0.0246 (13)	0.0008 (10)	−0.0021 (10)	0.0034 (10)
C10	0.0220 (14)	0.0198 (12)	0.0273 (13)	−0.0028 (10)	−0.0065 (10)	0.0044 (10)
C11	0.0302 (15)	0.0177 (11)	0.0236 (13)	−0.0019 (10)	−0.0045 (10)	−0.0018 (9)

### Geometric parameters (Å, º)

**Table:**

S1—C1	1.785 (3)	C3—H3B	0.9900
S1—C2	1.803 (2)	C4—H4A	0.9900
O1—C5	1.210 (3)	C4—H4B	0.9900
O2—C9	1.221 (3)	C5—C6	1.479 (3)
N1—C9	1.394 (3)	C6—C10	1.381 (3)
N1—C5	1.404 (3)	C6—C7	1.408 (3)
N1—C4	1.471 (3)	C7—C7^i^	1.413 (4)
C1—H1A	0.9800	C7—C8	1.415 (3)
C1—H1B	0.9800	C8—C11	1.373 (3)
C1—H1C	0.9800	C8—C9	1.478 (3)
C2—C3	1.523 (3)	C10—C11^i^	1.404 (3)
C2—H2A	0.9900	C10—H10	0.9500
C2—H2B	0.9900	C11—C10^i^	1.404 (3)
C3—C4	1.521 (3)	C11—H11	0.9500
C3—H3A	0.9900		
			
C1—S1—C2	100.17 (12)	N1—C4—H4B	108.9
C9—N1—C5	125.2 (2)	C3—C4—H4B	108.9
C9—N1—C4	118.31 (19)	H4A—C4—H4B	107.7
C5—N1—C4	116.52 (19)	O1—C5—N1	120.4 (2)
S1—C1—H1A	109.5	O1—C5—C6	122.9 (2)
S1—C1—H1B	109.5	N1—C5—C6	116.7 (2)
H1A—C1—H1B	109.5	C10—C6—C7	120.5 (2)
S1—C1—H1C	109.5	C10—C6—C5	119.5 (2)
H1A—C1—H1C	109.5	C7—C6—C5	120.0 (2)
H1B—C1—H1C	109.5	C6—C7—C7^i^	119.5 (2)
C3—C2—S1	110.75 (16)	C6—C7—C8	121.3 (2)
C3—C2—H2A	109.5	C7^i^—C7—C8	119.3 (3)
S1—C2—H2A	109.5	C11—C8—C7	120.0 (2)
C3—C2—H2B	109.5	C11—C8—C9	120.4 (2)
S1—C2—H2B	109.5	C7—C8—C9	119.6 (2)
H2A—C2—H2B	108.1	O2—C9—N1	120.2 (2)
C4—C3—C2	110.2 (2)	O2—C9—C8	122.6 (2)
C4—C3—H3A	109.6	N1—C9—C8	117.2 (2)
C2—C3—H3A	109.6	C6—C10—C11^i^	119.7 (2)
C4—C3—H3B	109.6	C6—C10—H10	120.1
C2—C3—H3B	109.6	C11^i^—C10—H10	120.1
H3A—C3—H3B	108.1	C8—C11—C10^i^	121.1 (2)
N1—C4—C3	113.37 (19)	C8—C11—H11	119.5
N1—C4—H4A	108.9	C10^i^—C11—H11	119.5
C3—C4—H4A	108.9		
			
C1—S1—C2—C3	−163.99 (17)	C6—C7—C8—C11	179.8 (2)
S1—C2—C3—C4	75.4 (2)	C7^i^—C7—C8—C11	−0.3 (4)
C9—N1—C4—C3	−84.9 (2)	C6—C7—C8—C9	−1.7 (3)
C5—N1—C4—C3	94.0 (2)	C7^i^—C7—C8—C9	178.1 (2)
C2—C3—C4—N1	−167.95 (19)	C5—N1—C9—O2	−178.5 (2)
C9—N1—C5—O1	176.4 (2)	C4—N1—C9—O2	0.3 (3)
C4—N1—C5—O1	−2.5 (3)	C5—N1—C9—C8	2.2 (3)
C9—N1—C5—C6	−4.1 (3)	C4—N1—C9—C8	−178.94 (19)
C4—N1—C5—C6	177.07 (18)	C11—C8—C9—O2	0.0 (3)
O1—C5—C6—C10	2.3 (3)	C7—C8—C9—O2	−178.4 (2)
N1—C5—C6—C10	−177.28 (19)	C11—C8—C9—N1	179.24 (19)
O1—C5—C6—C7	−177.5 (2)	C7—C8—C9—N1	0.8 (3)
N1—C5—C6—C7	3.0 (3)	C7—C6—C10—C11^i^	−0.4 (3)
C10—C6—C7—C7^i^	0.2 (4)	C5—C6—C10—C11^i^	179.8 (2)
C5—C6—C7—C7^i^	179.9 (2)	C7—C8—C11—C10^i^	0.5 (3)
C10—C6—C7—C8	−179.9 (2)	C9—C8—C11—C10^i^	−177.9 (2)
C5—C6—C7—C8	−0.2 (3)		

Symmetry code: (i) −*x*+2, −*y*+1, −*z*.

### Hydrogen-bond geometry (Å, º)

**Table:**

*D*—H···*A*	*D*—H	H···*A*	*D*···*A*	*D*—H···*A*
C4—H4*A*···S1	0.99	2.84	3.300 (2)	109
C4—H4*A*···O1	0.99	2.32	2.688 (3)	101
C10—H10···O1^ii^	0.95	2.39	3.316 (3)	164
C11—H11···S1^iii^	0.95	2.86	3.779 (2)	162

Symmetry codes: (ii) −*x*+1, −*y*, −*z*; (iii) *x*+1, *y*+1, *z*.
